# Neuropsychological profile associated with *KAT6A* syndrome: Emergent genotype-phenotype trends

**DOI:** 10.1186/s13023-024-03175-0

**Published:** 2024-05-13

**Authors:** Rowena Ng, Allison J Kalinousky, Jacqueline Harris

**Affiliations:** 1 Department of Neuropsychology, Kennedy Krieger Institute, 1750 E. Fairmount Ave, Baltimore, USA; 2 Department of Psychiatry and Behavioral Sciences, Johns Hopkins University School of Medicine, Baltimore, USA; 3 Department of Genetic Medicine, Johns Hopkins University School of Medicine, Baltimore, USA; 4 Department of Pediatrics, Johns Hopkins University School of Medicine, Baltimore, USA; 5 Department of Neurology, Johns Hopkins University School of Medicine, Baltimore, USA

**Keywords:** Genetics/Genetic disorders, KAT6A, Histone machinery, Epigenetic, Cognition, Behavior, Social functioning

## Abstract

**Background:**

*KAT6A* (Arboleda-Tham) syndrome is a Mendelian disorder of the epigenetic machinery caused by pathogenic variants in the lysine acetyltransferase 6 A (*KAT6A*) gene. Intellectual disability and speech/language impairment (e.g., minimally verbal) are common features of the disorder, with late-truncating variants associated with a more severe form of intellectual disability. However, much of the cognitive phenotype remains elusive given the dearth of research.

**Participants and methods:**

This study examined non-verbal and social skills of 15 individuals with molecularly-confirmed diagnoses of *KAT6A* syndrome (Mean age = 10.32 years, SD = 4.12). Participants completed select subtests from the DAS-II, the NEPSY-II, and the Beery Buktenica Developmental Test of Visual Motor Integration 6th Edition, and their caregivers completed an assortment of behavior rating inventories.

**Results:**

Findings suggest global cognitive impairment with nonverbal cognition scores similar to those for receptive language. Autism-related features, particularly restricted interests and repetitive behaviors, and broad adaptive deficits were common in our sample juxtaposed with a relatively strong social drive and low frequency of internalizing and externalizing behavioral problems. A general trend of lower performance scores on nonverbal and receptive language measures was observed among those with protein-truncating variants vs. missense variants; however, no effect was observed on caregiver rating inventories of daily behaviors. Late and early truncating variants yielded comparable neuropsychological profiles.

**Conclusions:**

Overall, study results show the cognitive phenotype of *KAT6A* syndrome includes equally impaired nonverbal cognition and receptive language functioning, paired with relatively intact social drive and strengths in behavior regulation. Emergent genotype-phenotype correlations suggest cognition may be more affected in protein-truncating than missense mutations although similar neurobehavioral profiles were observed.

## Introduction

*KAT6A* syndrome (Arboleda-Tham syndrome; MIM: 616,268) is a rare Mendelian disorder of epigenetic machinery (MDEM), a class of relatively newly defined neurodevelopmental disorders that result from mutations in genes dedicated to encoding epigenetic regulators [1]. *KAT6A* syndrome is caused by a pathogenic variant in histone K lysine acetyltransferase *KAT6A* (Arboleda et al., 2015), a gene that belongs to the MYST family of histone acetyltransferases that facilitates histone acetylation and regulation of transcription. The prevalence rate of this syndrome is not clear although 500 cases of *KAT6A* and *KAT6B* disorders have been reported through the patient-driven KAT6 Foundation (www.kat6a.org). Individuals with *KAT6A* syndrome, like other MDEMs, share some overlapping traits including intellectual disability, hypotonia, and congenital heart defects [2, 3]. Other cardinal features of *KAT6A* disorder include microcephaly, unique facial dysmorphology, vision defects like strabismus and ptosis, oromotor dysfunction, hypotonia, gastrointestinal issues, congenital cardiac malformation, and sleep disturbance Arboleda et al., 2015 [2–5].

The neuropsychological profile of *KAT6A* syndrome remains relatively unknown although intellectual disability and/or global developmental delay is almost universally seen among affected individuals [6]. Language is severely affected, with nearly 75% of affected individuals presenting with minimally verbal skills [6]. Previously, receptive language or comprehension of language was reported to be more preserved [2], however, recent findings have shown receptive language is similarly affected in those with this syndrome [6]. Investigations on genotype-phenotype correlations in cognition and neurobehavioral functioning in *KAT6A* syndrome have yielded mixed results. Late truncating variants (exons 16–17) as compared to early truncating (exons 1–15) have been associated with more severe intellectual disability based on clinician-ratings or documented diagnoses based on medical records [2, 6] and greater difficulties in receptive communication, socialization, and daily living skills based on parent-rating inventories [6]. Other prospective behavioral studies did not observe differences in internalizing, externalizing, and adaptive behaviors based on truncating variant [4]. Notably, a vast majority of studies focused on *KAT6A* syndrome rely on retrospective review of medical charts and/or clinician- or caregiver-rating measures rather than performance-based testing. Other than language [6], it remains unclear the extent other cognitive domains may be affected in these individuals. Likewise, it is possible the documented rates of intellectual disability associated with *KAT6A* syndrome may largely reflect verbal/language deficits whereby nonverbal skills are more preserved.

Accordingly, this study aimed to prospectively characterize nonverbal cognition and neurobehavioral functioning in those with *KAT6A* syndrome. Genotype-phenotype correlations (truncating vs. missense variants) were examined. To our knowledge, this study is the first in the literature to define the neuropsychological phenotype of *KAT6A* syndrome utilizing a combination of standardized performance-based and caregiver-report measures. Given the exploratory nature of this investigation, we had no a priori hypotheses.

## Methods

### Participants

A total of 15 individuals with *KAT6A* syndrome participated in this study (8 F, Mean age = 10.32 years, SD = 4.12, range = 4–20). Our sample was largely non-Hispanic, White (80%). As shown in Table 1, demographic background was similar between those with truncating vs. missense variant. All participants were recruited through the KAT6 Foundation via their website or social media platforms (Twitter, Facebook).

**Table 1:** Participant characteristics and average ratings on caregiver-rating inventories

	Truncating Variant (*N* = 12)	Missense Variant (*N* = 3)	Whole Sample (*N* = 15)	Mann Whitney U or Fishers Exact Test, p-value (truncating vs. missense variants)
Sex	6 F	2 F	8 F	n.s.
Mean Age in Years(SD)[range]	14.66(4.96) [2–11]	9.24(3.27) [1, 7–10, 12–17]	10.32(4.12) [1–17]	n.s.
Race				
White	86.67%	66.67%	80%	n.s.
Asian	0%	33.33%	13.33%	
Multiracial	13.33%	0%	6.67%	
Intervention History (% of sample that participated in therapy)				
Speech/language Therapy	100%	100%	100%	n.s.
Occupational Therapy	100%	100%	100%	n.s.
Physical Therapy	91.67%	66.67%	86.67%	n.s.
Behavior Therapy	8.33%	66.67%	20%	FET = 5.10, *p* = 0.08
Diagnostic history as reported by caregiver (% of sample)				
Intellectual Disability	90.90%	66.67%	85.71%	n.s.
Autism Spectrum Disorder	27.27%	33.33%	28.57%	n.s.
Attention Deficit Hyperactivity Disorder	27.27%	33.33%	28.57%	n.s.

*Note* n.s. = not significant (p-value > 0.10). One caregiver did not complete the diagnostic history section of the research intake questionnaire, thus, percentage of sample is computed of *N* = 14

**Fig. 1:**
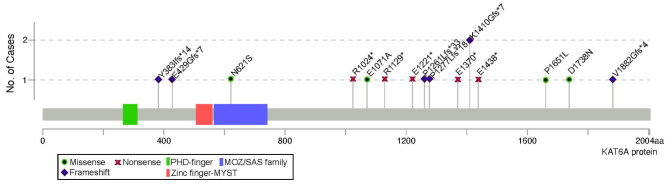
Schematic diagram of the pathogenic variants identified in our clinical sample. *Note*. Participant #4 has both variants p.N621S and p.P1651L

A physician at the authors’ institute reviewed genetic test records shared by caregivers to confirm the variant in *KAT6A*. Of the 15 participants, 12 had a truncating variant (2 with early truncating variants in exons 1 to 15, 10 with late truncating variants in exon 16 and 17), and three had missense variants. The majority of the sample were diagnosed through whole exome sequencing (73.33%) while the remainder were diagnosed from genetic panel testing. Most were classified as a pathogenic variant (86.67%). One had a variant of uncertain significance but has been examined by an author on this paper and the clinical phenotype is consistent with *KAT6A* syndrome, and another possessed a likely pathogenic variant. Most variants were de novo (73.33%), while three had unknown inheritance as parents were not tested. Figure 1 illustrates the *KAT6A* variants in our clinical sample.

### Procedure

Prior to the assessment appointment, all caregivers completed a research intake questionnaire that inquired about their child’s developmental history, intervention history (i.e., current and past engagement in therapies), and diagnostic history of intellectual disability, autism spectrum disorder, and attention deficit/hyperactivity disorder (ADHD). One parent did not complete the form in full and thus diagnoses were not documented. Cognitive testing was completed at the annual KAT6 Foundation Conference held in Boston, USA (*N* = 9), or at the Department of Neuropsychology at Kennedy Krieger Institute (*N* = 6). Parents of participants completed additional standardized caregiver-report questionnaires at the time of testing.

### Materials

#### Parent-rating inventories

All inventories (outlined below) are rated on a Likert-scale with responses normed by age and sex, when available. The Behavior Rating Inventory of Executive Functioning 2nd Edition (BRIEF-2) [8], Preschool version (BRIEF-P) [7] or Adult version (BRIEF-A) [11] were administered to index daily executive functioning. The inventories provide several subscales of executive functions – those that overlap across the three versions include Inhibit, Emotional Control, Working Memory, Planning/Organize, and Shift. Inhibit refers to impulse control and behavior disengagement, while Working Memory represents the ability to hold information and/or manipulate it in goal-directed behaviors. Emotional Control indexes emotion regulation and Shift represents flexibility in problem-solving or transitioning between activities. Planning/Organize refers to the ability to develop a plan, set objectives, and work towards the targeted goal. All three versions also yield a Global Executive Composite that daily problems with executive functioning. Of note, only one participant was an adult and thus her parent completed the BRIEF-A. On the BRIEF, T-Scores ≥ 70 represent clinically significant problems in the domain, and scores of 60–69 denote areas at risk.

The Social Responsiveness Scale 2nd Edition (SRS-2) [15] is an inventory often used to screen for behaviors related to autism spectrum disorder (ASD). The five domains indexed by the SRS-2 include Social Awareness, Social Cognition, Social Communication, Social Motivation, and Restricted Interests and Repetitive Behaviors (RRB); and these together yield the SRS-2 Total Composite. Social Awareness represents ones’ sensitivity to detect social cues while Social Cognition indexes the extent one can interpret social cues. Social Communication represents reciprocal communication in interactions, and Social Motivation measures day-to-day motivation to engage in social interactions with others. RRB refers to observed stereotypies and highly restricted interests. T-scores of 60–65 indicate mild difficulty, 66–75 indicate moderate difficulty, and 76 or above indicates severe challenges.

The Child Behavior Checklist (CBCL) is a rating measure used to assess behavioral problems [18–20]. This measure yields Internalizing and Externalizing Behaviors scales, as well as a Social Problems subscale. Internalizing Behaviors scale consists of items about anxious/low mood and withdrawn behaviors, while Externalizing Behaviors scale includes noncompliant, aggressive and rule-breaking tendencies. Social Problems subscales index daily challenges in peer interactions and relationships. T-Scores ≥ 70 represent clinically significant problems in the area.

Daily adaptive behaviors were assessed by the Adaptive Behavior Assessment 3rd Edition (ABAS-3) [9]. The ABAS-3 requires caregivers to rate their child’s adaptive behaviors across three domains (Conceptual, Social, Practical). The Conceptual domain comprises of Functional Communication, Functional Academics, and Self-Direction. The Social domain includes both Social and Leisure scales. The Practical domain is computed by the following scales Community Use, Home Living, Health and Safety, and Self-Care. All scales are needed to yield the General Adaptive Composite (GAC), a measure of overall adaptive functioning. Domains that yield standard scores < 70 are extremely low or impaired.

Of note, standard scores and T-scores have means of 100 and 50, and standard deviations of 15 and 10 respectively. Elevated T-scores across the BRIEF, CBCL, and SRS-2 are indicative of more difficulty in the behavioral domain, whereas lower standard scores on the ABAS-3 reflect more challenges in the adaptive skill area.

#### Performance-based cognitive measures

The following cognitive tests were selected to form a brief test battery that could be administered within 1.5 hours at an annual family conference for those affected by *KAT6A* syndrome (KAT6 Conference). Initially, test measures were selected for school- to adolescent-age youth. One adult participant later enrolled as the study recruitment continued after the conference. DAS-II and NEPSY-II were not administered to that individual given she was out of the available age norms for the tests.

Based on the participant’s age at testing, the Early Years or School-Age versions of the Differential Ability Scale 2nd Edition (DAS-II) [17] was administered. Those between 7 years to 8 years, 11 months were administered the Early Years version given the low performance on receptive language measures. Past studies have highlighted severe expressive language difficulties among individuals with *KAT6A* syndrome with most presenting with limited to minimally verbal skills [6], as such, non-verbal cognition was emphasized in this study. Both versions offer a Special Nonverbal Composite based on performance across four subtests. In the Early Years version, this composite was comprised of Pattern Construction, Picture Similarities, Matrices and Copying subtests. In the School-Age version. Pattern Construction, Matrices, Recall of Designs and Sequential and Quantitative Reasoning subtests were the main non-verbal measures.

Based on the participant’s age, Arrows and Comprehension of Instructions subtests from the Developmental Neuropsychological Assessment (NEPSY-II) [14] were used to assess visuospatial perception and receptive language respectively. These subtests were included in our test battery as these they do not require verbal responses. Participants are allowed to point or touch pictures to answer test items. NEPSY-II can be used to assess children between age 3 to 16, although specific subtests vary in available age-based norms. In line with the test manual, NEPSY-II Comprehension of Instructions was given to individuals aged 3 to 16 years, while NEPSY-II Arrows was administered to those aged 5 to 16 years.

To measure visuomotor and visual perceptual skills, the Beery-Buktenica Developmental Test of Visual-Motor Integration 6th Edition (VMI-6) [12] was included in the research assessment. This test can be administered to individuals aged 2 years and older. The Visual Perceptual and Visual-Motor Integration subtests were selected to examine visual matching of geometric shapes and eye-hand coordination when copying illustrations of shapes of increasing complexity.

Finally, the Peabody Picture Vocabulary Test 5th Edition (PPVT-5) [16], which assesses receptive vocabulary in individuals aged 2.5 years and older, was included in testing. All performance raw scores were converted to standard scores using age norms provided in test manuals. Lower standard scores reflect more difficulty in the cognitive measure.

### Data strategy

Descriptive analyses were used to examine the proportion of participants with a diagnosis of intellectual disability, ASD, and ADHD; and current or prior participation in interventions (speech/language, occupational, physical, behavioral therapies).

With caregiver-report measures, we examined the proportion of our sample with impairment in behavioral functioning (standard score of < 70 on the ABAS-3, T-score > 70 on the BRIEF, SRS-2, or CBCL). Friedman test was used to determine within-group differences across BRIEF, ABAS, CBCL, and SRS-2 Scales in the whole sample to identify areas of relative strengths/weaknesses. Pairwise comparisons with Bonferroni correction were subsequently applied given multiple tests. Although our sample of participants with missense variants is small, Mann-Whitney U-test was utilized to provide preliminary findings on genotype-phenotype associations across behavioral domains, which can be used to inform future investigations.

With cognitive assessment measures, we similarly applied Friedman Test to determine within-group differences in performance across DAS-II Special Nonverbal Composite, PPVT-5, and NEPSY-II Comprehension of Instruction. VMI-6 was not included in this analysis given the DAS-II subtests that comprise the Special Nonverbal Composite measure some similar constructs (e.g., VMI-6 Visual-Motor Integration and DAS-II Copying both assess ones’ ability to draw or copy simple designs). NEPSY-II Arrows was also not included in this analysis, as we were selective in choosing cognitive tests given the limited sample size. Mann-Whitney U-test was repeated to identify any effect of truncating variants on cognitive domains. Of note, given limited individuals with early truncating variants, we first examined broad differences in behavioral and cognitive functioning between those with truncating vs. missense variants. Areas that yielded significant effect of truncating variants (truncating vs. missense variants) were then subject to Kruskal-Wallis test with three groups (early truncating, late truncating, missense variant).

Finally, given a relatively high number of individuals who were unable to participate in testing across measures due to limited comprehension skills (e.g., unable to demonstrate understanding of task instructions with practice items and feedback), we examined the extent these participants were comprised of those with late/early truncating vs. missense variant.

## Results

### Diagnostic history

Of the 14 parents who completed the diagnostic history section of our research intake questionnaires, 12 reported their child has a diagnosis of intellectual disability (85.71%), 4 with ASD (28.57%), and 4 with ADHD (28.57%). All 14 participants had at least one of these three diagnosis. Those with a truncating vs. missense variants did not show significant difference in diagnostic history for the neurodevelopmental disorders (Table 1).

### Intervention history

The entire sample reported a history of occupational and speech language therapy, and most have a history of physical therapy (86.67%). In contrast, only 26.67% reported a history of behavior therapy. The proportion of the participants with a history of behavior therapy was slightly more robust in those with missense variants than those with truncating variants (Table 1).

### Parent-rating inventories

Table 2 outlines mean ratings and proportion of our whole sample that reached clinical significance on behavioral measures, which was defined as two standard deviations from the normative mean. Table 3 shows the average ratings across inventory among those with truncating/missense variants. No effect of variant type (truncating, missense variant) was observed across inventories.

**Table 2:** Proportion of sample with clinically significant behavioral functioning difficulties across the Behavior Rating Inventory of Executive Function (BRIEF), Child Behavior Checklist (CBCL), Social Responsiveness Scale 2nd Edition (SRS-2), and Adaptive Behavior Assessment Scale 3rd Edition (ABAS-3)

	Mean(Standard Deviation)[range]	Percentage of sample significantly deviant (≥ 2SD) from normative mean
**BRIEF, T-Score**		
Emotional Control	55.18(7.52)[43–66]	0%
Inhibit	65.45(3.61)[61–72]	18.18%
Shift	65.73(10.91)[49–79]	45.45%
Working Memory	70.18(8.64)[50–83]	72.72%
Planning/Organize	61.64(6.87)[47–74]	9.09%
Global Executive Composite	67.64(3.66)[61–73]	36.36%
**CBCL, T-Score**		
Internalizing Behaviors	57.93(9.34)[45–73]	14.28%
Externalizing Behaviors	51.21(8.48)[33–66]	0%
Social Problems	62.85(7.95)[52–79]	15.38%
**SRS-2, T-Score**		
Social Awareness	68.07(9.86)[51.82]	53.33%
Social Cognition	68.80(11.02)[49–85]	46.67%
Social Communication	69.40(11.74)[51–86]	46.67%
Social Motivation	61.13(10.00)[44–75]	20.00%
Restricted Interests/ Repetitive Behaviors	73.67(14.18)[41–90]	66.67%
Total	71.40(9.70)[55–84]	60.00%
**ABAS-3, Standard Score**		
Conceptual	61.93(11.78[48–93]	86.67%
Social	68.13(12.13)[52–96]	53.33%
Practical	62.67(14.45)[48–88]	73.33%
General Adaptive Composite	61.27(13.17)[46–90]	73.33%

*Note* The proportion of our sample with impairment in behavioral functioning refers to the participants with standard score of < 70 on the ABAS-3, or T-score > 70 on the BRIEF, CBCL, or SRS-2

**Table 3:** Participant characteristics and average ratings on caregiver-rating inventories

	Truncating Variant (N_total_=12)			Missense Variant (N_total_=3)	Mann Whitney U, p-value (truncating vs. missense variants)
**Mean (SD)[range]**	Late Truncating	Early Truncating	Truncating Variant Total		
**BRIEF, T-Score**	*N* = 6	*N* = 2	*N* = 8	*N* = 3	
Emotional Control	53.83(6.85)[43–61]	61.00(5.65)[57–65]	55.63(7.00)[43–65]	54.00(10.39)[48–66]	n.s.
Inhibit	65.83(4.95)[61–72]	65.50(0.70)[65–66]	65.75(4.20)[61–72]	64.67(1.52)[63–66]	n.s.
Shift	63.50(10.59)[52–79]	72.50(9.19)[66–79]	65.75(10.47)[52–79]	65.67(14.57)[49–76]	n.s.
Working Memory	69.67(10.32)[50–79]	67.00(4.24)[64–70]	69.00(8.96)[50–79]	73.33(8.50)[67–83]	n.s.
Planning/Organize	61.83(8.63)[47–74]	63.50(7.77)[58–69]	62.25(7.90)[47–74]	60.00(3.46)[58–64]	n.s.
Global Executive Composite	67.17(4.83)[61–73]	70.00(1.41)[69–71]	67.88(4.32)[61–73]	67.00(1.00)[66–68]	n.s.
**CBCL, T-Score**	*N* = 10	*N* = 2	*N* = 12	*N* = 2	
Internalizing Behaviors	58.10(9.49)[45–73]	59.00(15.55)[48–70]	58.25(9.79)[45–73]	56.00(8.48)[50–62]	n.s.
Externalizing Behaviors	51.30(5.98)[44–61]	49.50(23.33)[33–66]	51.00(8.90)[33–66]	52.50(7.77)[47–58]	n.s.
Social Problems	63.56(7.56)[53–79]	67.50(10.60)[60–75]	64.27(7.72)[52–79]	55.00(4.24)[52–58]	n.s.
**SRS-2, T-Score**	*N* = 10	*N* = 2	*N* = 12	*N* = 3	
Social Awareness	66.30(10.75)[51–82]	73.50(12.02)[65–82]	67.50(10.74)[51–82]	70.33(6.02)[64–76]	n.s.
Social Cognition	69.80(10.66)[50–83	76.00(12.72)[67–85]	70.83(10.65)[50–85]	60.67(10.11)[49–67]	n.s.
Social Communication	69.00(13.03)[51–86]	72.50(7.77)[67–78]	69.58(12.10)[51–86]	68.67(12.58)[57–82]	n.s.
Social Motivation	61.80(8.82)[48–75]	65.50(4.95)[62–69]	62.42(8.25)[48–75]	56.00(16.64)[44–75]	n.s.
Restricted Interests/ Repetitive Behaviors	73.50(16.46)[41–90]	80.00(2.82)[78–82]	74.58(15.12)[41–90]	70.00(11.13)[60–82]	n.s.
SRS-2 Total	71.60(9.70)[58–84]	76.00(8.48)[70–82]	72.33(9.30)[58–84]	67.67(12.50)[55–80]	n.s.
**ABAS-3, Standard Score**	*N* = 10	*N* = 2	*N* = 12	*N* = 3	
Conceptual	61.80(13.69)[48–93]	60.50(10.60)[53–68]	61.58(12.80)[48–93]	63.33(8.14)[54–69]	n.s.
Social	68.90(14.49)[52–96]	66.00(9.89)[59–73]	68.42(13.49)[52–96]	67.00(5.19)[64–73]	n.s.
Practical	61.70(14.11)[48–88]	54.50(0.70)[54–55]	60.50(13.07)[48–88]	71.33(19.50)[49–85]	n.s.
General Adaptive Composite	60.90(14.76)[46–90]	56.50(6.36)[52–61]	60.17(13.59)[46–90]	65.67(12.70)[51–73]	n.s.

*Abbreviation* n.s. = not significant

#### Child behavior checklist (CBCL)

Wilcoxon signed rank test showed caregivers provided more elevated ratings for Internalizing than Externalizing behaviors (Z=-3.08, *p* = 0.002). Despite this trend, on average, our sample was rated within broad typical range for Internalizing and Externalizing behaviors, and Social Problems – a pattern that was observed across truncating and missense variants (Table 3). Approximately 15% of the sample was rated to present with clinical level of Internalizing behaviors or Social Problems, but no participant met clinical cut-off for Externalizing behaviors.

#### Behavior rating inventory of executive functioning (BRIEF-P, BRIEF-2, BRIEF-A)

Overall executive functioning, indexed by BRIEF Global Executive Composite, was in the at-risk range (Table 2) with 36% of individuals in the clinical range for global executive composite problems. Within-group comparisons indicate executive functions are differentially affected in those with *KAT6A* syndrome (χ^2^(4) = 19.83, *p* < 0.001). Emotional Control yielded less elevated ratings than Inhibit (*p* = 0.05), Shift (*p* = 0.006), or Working Memory (*p* = 0.002). Across subscales, Working Memory was the only area that met clinical level of difficulty, and while Emotional Control was the one executive function that was within typical range. Over 70% of the sample present with significant difficulties in Working Memory, whereas none of the participants was rated to show challenges with Emotional Control. A sizeable proportion of our sample also demonstrates clinical level of problems with Shift, or the ability to problem solve flexibly (45%).

#### Social Responsiveness Scale 2nd Edition (SRS-2)

Per parent ratings, our sample of individuals with *KAT6A* syndrome present with moderate severity of ASD-related features (Table 2). Within-group comparisons revealed lower scores in Social Motivation in our sample than Social Communication (*p* = 0.07) and Restricted Interests/Repetitive Behavior (RRB)(*p* = 0.005)(χ^2^(4) = 13.67, *p* = 0.008). Specifically, Social Motivation was rated in the mild severity range whereas all other domains were rated in the moderate level. Likewise, while 20% of our sample yielded Social Motivation scores that were two standard deviations above the normative mean, over twice as many participants were rated to demonstrate prominent problems in other domains (Social Cognition, Social Communication, Social Awareness, RRB). In contrast, two-thirds of our sample met this cut-off for RRB. In brief, lack of social drive is a less common feature in this syndrome, whereas RRB may be a prominent disease characteristic.

#### Adaptive Behavior Assessment 3rd Edition (ABAS-3)

General Adaptive Composite was very low in our sample. Although, Conceptual, Social and Practical domains were all rated in the very low range, individuals with *KAT6A* syndrome show different levels of challenge across these areas (χ^2^(2) = 12.31, *p* = 0.002) (Table 2). Conceptual and Practical domains were rated lower than Social domain (*p* = 0.003 and 0.04 respectively). Approximately half our sample was rated over two standard deviations below the normative mean for the Social domain compared to over 70% in Practical and Conceptual domains.

In summary, across rating inventories, those with truncating vs. missense variants generally presented with similar behavioral profiles. Areas that are of relative strength for those with *KAT6A* syndrome include emotion control, behavior regulation, and an affinity for socially interacting with others.

### Cognitive testing

Table 4 outlines the average performance across cognitive measures. In the whole sample of individuals with *KAT6A* syndrome, participants performed significantly below normative mean (> 2 standard deviations) across virtually all measures with the exception of NEPSY-II Arrows which assesses spatial perception. Friedman test did not reveal differences in performance scores across cognitive measures, suggesting non-verbal cognition is similarly affected as receptive language skills (χ^2^(2) = 3.80, *p* = 0.15).

**Table 4:** Mean performance scores across cognitive measures. Standard deviation in parentheses

Cognitive Tests, Standard Scores	Truncating Variant (*N* = 12)			Missense Variant (*N* = 3)	Whole Sample (*N* = 15)	Mann Whitney U, p-value (truncating vs. missense variant)
	Late Truncating Variant (*N* = 10)	Early Truncating Variant (*N* = 2)	All Truncating Variants (*N* = 12)			
**Nonverbal Skills**						
DAS-II Special Nonverbal Composite	*N* = 7, 48.43(17.46)	*N* = 2, 51.50(30.40)	*N* = 9, 49.11(18.60)	*N* = 2, 78.50(4.95)	*N* = 11, 54.45(20.51)	U = 0.50, *p* = 0.036
DAS-II Pattern Construction	*N* = 7, 54.85(15.58)	*N* = 2, 62.50(31.81)	*N* = 9, 56.55(17.88)	*N* = 2, 94.00(8.48)	*N* = 11, 62.36(22.19)	U = 0.50, *p* = 0.036
DAS-II Matrices	*N* = 7, 69.71(13.40)	*N* = 2, 64.50(34.64)	*N* = 9, 68.55(17.03)	*N* = 2, 80.00(0.00)	*N* = 11, 70.63(15.92)	n.s.
NEPSY-II Arrows	*N* = 5, 69.00(19.17)	*N* = 2, 75.00(28.28)	*N* = 7, 70.71(19.66)	*N* = 2, 80.00(35.35)	*N* = 9, 72.77(21.52)	n.s.
Beery Visual Perception	*N* = 7, 64.14(19.20)	*N* = 2, 65.00(28.28)	*N* = 9, 64.33(19.41)	*N* = 2, 88.00(9.89)	*N* = 11, 68.64(20.07)	n.s.
Beery Visual motor Integration	*N* = 7, 49.00(6.95)	*N* = 2, 61.00(22.62)	*N* = 9, 51.67(11.32)	*N* = 3, 56.33(10.26)	*N* = 12, 52.83(10.81)	n.s.
**Receptive Language**						
PPVT-5	*N* = 7, 61.00(11.70)	*N* = 2, 62.50(31.82)	*N* = 9, 61.33(15.15)	*N* = 3, 59.00(25.51)	*N* = 12, 60.75(16.92)	n.s.
NEPSY-II Comprehension of Instructions	*N* = 7, 60.71(9.32)	*N* = 2, 57.50(3.53)	*N* = 9, 60.00(8.29)	*N* = 2, 75.00(28.28)	*N* = 11, 62.72(13.10)	n.s.

*Abbreviations* DAS-II = Differential Abilities Scale 2nd Edition, PPVT-5 = Peabody Picture Vocabulary Test 5th Edition, NEPSY-II = A Developmental Neuropsychological Assessment 2nd Edition, n.s. = not significant

Note One participant (20-year-old) with missense variant was out of age range for DAS-II and NEPSY-II. This participant was not counted in sample proportions above as lack of participation was not attributed to comprehension impairment

**Fig. 2:**
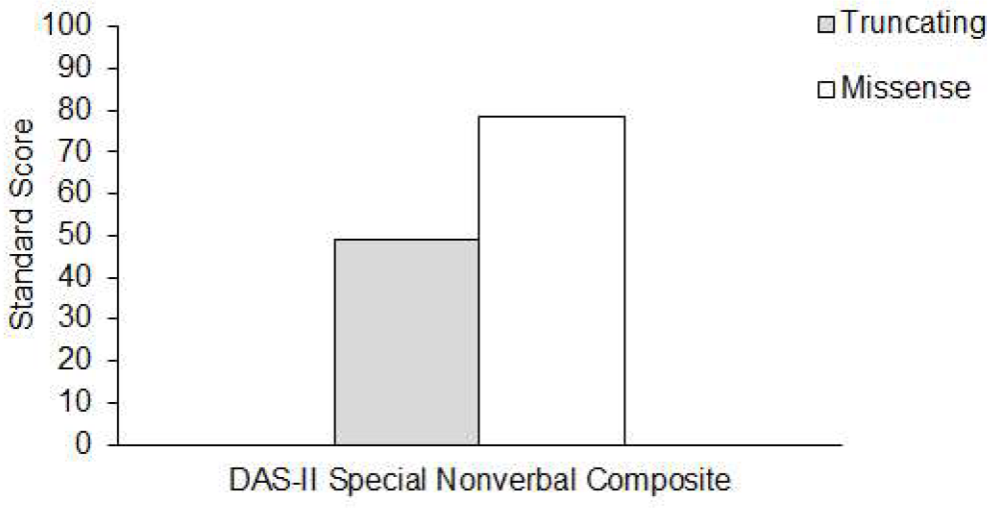
Mean non-verbal composite scores across participants with variants in *KAT6A*

Descriptive analyses also generally show a trend towards higher performance across nearly all cognitive measures among those with a missense variant vs. a protein-truncating variant. However, with inferential statistics, limited cognitive tests yielded an effect of variant type. Those with a truncating variant yielded lower DAS-II Special Nonverbal Composite, which represents non-verbal skills broadly (Table 4) (Fig. 2) largely due to their low performance scores on the DAS-II Pattern Construction subtest, which assesses visuomotor skills (Fig. 2). A marginal effect of variant type was found in performance scores on the DAS-II Pattern Construction (χ^2^(2) = 4.64, *p* = 0.09) with a trend of stronger visuomotor skills in those with a missense variant than late truncating variant (Table 4). As shown in Table 5, compared to the early truncating and missense groups, a large proportion of those with late truncating variants were unable to complete cognitive test measures due to comprehension difficulties (e.g., unable to demonstrate understanding of task instructions with practice items and feedback or modified directions).

**Table 5:** Proportion of the subsamples who could not test due to comprehension deficits

Cognitive Test (Participants who were unable to complete test)	Late Truncating Variant (*N* = 10)	Early Truncating Variant (*N* = 2)	Missense Variant (*N* = 3)
**Nonverbal Skills**			
DAS-II Pattern Construction (*N* = 3)	3 of 10 (30%)	0 of 2 (0%)	0 of 2 (0%)
DAS-II Matrices (*N* = 3)	3 of 10 (30%)	0 of 2 (0%)	0 of 2 (0%)
NEPSY-II Arrows (*N* = 5)	5 of 10 (50%)	0 of 2 (0%)	0 of 2 (0%)
Beery Visual Perception (*N* = 4)	3 of 10 (30%)	0 of 2 (0%)	1 of 3 (33%)
Beery Visual motor Integration (*N* = 3)	3 of 10 (30%)	0 of 2 (0%)	0 of 3 (0%)
**Receptive Language**			
PPVT-5 (*N* = 3)	3 of 10 (30%)	0 of 2 (0%)	0 of 3 (0%)
NEPSY-II Comprehension of Instructions (*N* = 3)	3 of 10 (30%)	0 of 2 (0%)	0 of 2 (0%)

*Abbreviations* DAS-II = Differential Abilities Scale 2nd Edition, PPVT-5 = Peabody Picture Vocabulary Test 5th Edition, NEPSY-II = A Developmental Neuropsychological Assessment 2nd Edition

Note One participant (20-year-old) with missense variant was out of age range for DAS-II and NEPSY-II. This participant was not counted in sample proportions above as lack of participation was not attributed to comprehension impairment

**Fig. 3:**
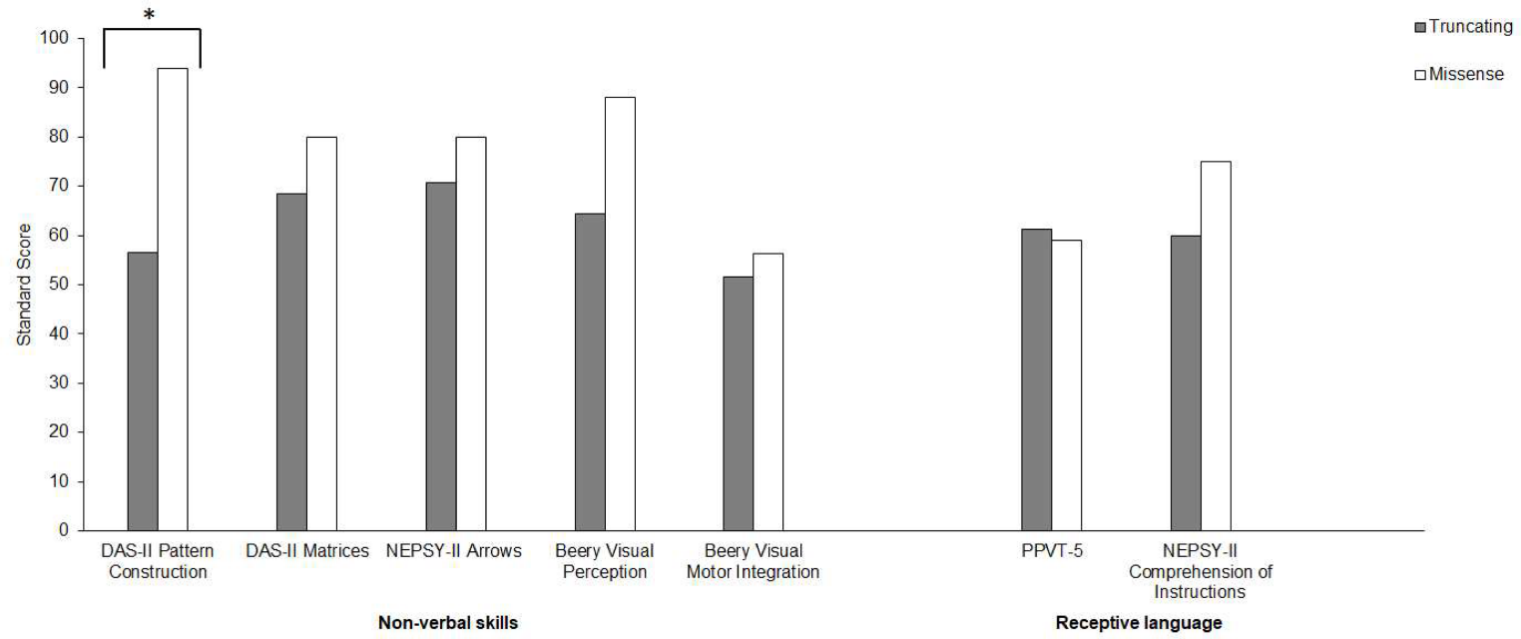
Mean performance scores across visuomotor, visual-spatial processing, receptive language, and social cognitive measures as a function of *KAT6A* variants

In brief, individuals with *KAT6A* syndrome present with global cognitive challenges that encompass both non-verbal skills and receptive language. Limited differences were observed between those with truncating and missense variants, albeit our low sample size reduces the statistical power to detect more nuanced effects (See Fig. 3).

## Discussion

To our knowledge, this study is the first study to use performance-based cognitive measures to characterize the neuropsychological phenotype of *KAT6A* syndrome. Main findings from our clinical sample highlight non-verbal cognition among those with *KAT6A* syndrome may be impaired akin to receptive language. Caregiver ratings also indicate global deficits in adaptive behaviors and high rates of inflexible behaviors in the context of relative strengths in emotion regulation and their desire to interact with others. Based on review of descriptive results, a trend of lower performance scores was observed among those with protein-truncating variants compared to those with missense variants albeit, when non-parametric tests were applied, this effect of variant type reached statistical significance on a very limited subset of the cognitive measures.

Among individuals with *KAT6A* syndrome, non-verbal reasoning skills and receptive language are not spared and similarly affected. While our abbreviated test battery did not include expressive language measures for comparison, our findings combined with recent findings on language communication skills in *KAT6A* syndrome [6] suggest global cognitive challenges, highlighting the central regulatory role *KAT6A* has on neurodevelopmental processes [21]. Clinicians who provide care management for those with *KAT6A* syndrome should consider interventions to support daily functioning for affected individuals beyond speech/language impairment.

Consistent with Smith and Harris [4], our sample of individuals with *KAT6A* syndrome demonstrate low rates of behavioral problems and relatively strong social drive, juxtaposed with adaptive deficits and significant restricted interests and repetitive behaviors. Those with truncating and missense variants shared this common neurobehavioral phenotype as well as similar participation in intervention services (speech/language, occupational, physical therapies). It is possible frontal-limbic neural networks may be differentially impacted by mutations in *KAT6A* compared to other neural systems, particularly given the frontal lobe undergoes protracted development postnatally. Longitudinal investigations that incorporate both cognitive testing and neurobiological methods (e.g., electroencephalogram, functional magnetic resonance imaging, magnetic resonance imaging) are necessary to determine if the function of the gene may impact specific neural substrates differentially. These efforts may aid in understanding the long-term regulatory impact this gene has on both structural and functional brain development.

Our study results, with small sample sizes, provided initial clues that truncating variants causing *KAT6A* syndrome may be associated with more difficulty in a measure of visuomotor skills than those with a missense variant. Notably, the performance scores across the vast majority of cognitive tests were higher in those with missense than protein-truncating mutations, although these additional measures did not reach statistical significance. Those with late truncating variants represented most of the participants who could not complete cognitive testing secondary to severe comprehension challenges. It is important to highlight our small sample sizes significantly limited our ability to ascertain more moderate effects in performance scores across truncating and missense groups, which may explain the few significant findings resultant from inferential analyses in spite of the consistent descriptive patterns. In brief, taken together, these observations offer initial clues that protein-truncating variants may be associated with more cognitive difficulties as compared to non-truncating variants. Given extremely small sample sizes in the early truncating and missense variant groups and lack of effects seen in all other visuomotor measures (e.g., Beery Visual Motor Integration), it is not possible to make conclusions on how genotype correlates with non-verbal cognition, but further investigations are warranted given the very low statistical power.

Interestingly, those with late truncating, early-truncating, and missense variants demonstrate comparable adaptive functioning deficits (Table 3). Although those with protein truncating variants yielded lower mean performance scores than those with missense variants, those with late and early truncating variants performed similarly across most cognitive tests. Overall, these patterns are in contrast with findings reported in Kennedy et al. [2] and St. John et al. [6], both suggesting severity of intellectual disability differs between those with late and early truncating variants in *KAT6A*. It should be noted that both studies measured intellectual disability by a review of medical records and/or clinician ratings. In addition, the rating criterion were not provided in the original published study. In all, severity of impairment was operationalized in a heterogeneous manner. Moreover, these studies did not provide detail in the manner intellectual functioning was determined. Given severe expressive language impairment commonly seen in individuals with *KAT6A* syndrome, if intelligence was assessed by full-scale intellectual quotient (FSIQ), which incorporates both verbal and non-verbal reasoning skills, IQ may be disproportionately affected by low performance scores on measures requiring verbal responses. Consequently, discrepancies between our results with prior investigations may stem from study design. Future investigations with a comprehensive cognitive test battery (e.g., including measures for expressive language), more robust participant recruitment, and integration of neurobiological metrics will be important to identify genotype-phenotype associations in *KAT6A* syndrome, particularly in neurocognition. Alternative methods to index cognitive functions (e.g., eye gaze) should be considered given higher rates of sensorimotor impairment among affected persons.

### Study limitations and future directions

Despite the novel contribution our study findings add to the sparse literature on neurocognition and *KAT6A* syndrome, future research should consider our methodological limitations in their study design. Caregivers provided diagnostic history in our study. Our intake form did not require parents to document the type of assessor (e.g., pediatrician, neurologist, neuropsychologist) or evaluation that yielded the diagnoses, and as such, heterogeneous clinical approaches were likely used in the classification of the developmental disorders. As evidenced by the number of participants who were unable to engage in select measures, more sensitive measurement tools that capture a wider range of cognition are needed. Other than traditional paper and pencil neuropsychological tests, use of eye-tracking and other methodological approaches that reduces motor demands may be important to consider, particularly when working with clinical populations with more medical complexities and significant hypotonia like KAT6 disorders. Our sample had a wide age range due to challenges with participant recruitment of ultra-rare diseases like *KAT6A* syndrome. Ideally, future studies should consider focusing on a cohort around the same developmental period. Finally, cross-syndrome research that compares neuropsychological profiles between those with *KAT6A* syndrome with other MDEMs with similar affected proteins (e.g., *KAT6B* disorders) or epigenetic machinery more broadly (disorder of the histone machinery such as Kabuki syndrome) will shed light on shared pathogenic pathways and downstream effects on neurodevelopment. Given animal models of MDEMs of the histone machinery like Rubinstein-Taybi syndrome have shown promise in the amelioration of cognitive dysfunction [13], detailed characterization of the phenotype associated with *KAT6A* syndrome and shared features with other MDEMS are critical steps towards determining outcome markers in designing clinical trials. From a clinical standpoint, these efforts will inform professionals who work with affected individuals to offer disease-specific care management and behavioral interventions.

## Conclusions

In summary, study results highlight the neuropsychological profile of *KAT6A* syndrome includes equally impaired nonverbal cognition similar to receptive language, high rates of inflexible behaviors, and global adaptive deficits, combined with relative strengths in emotion regulation and strong appetitive social drive. A preliminary review of genotype-phenotype trends showed nonverbal cognition, particularly visuomotor skills, may be slightly stronger among those with missense than protein-truncating variants, but investigations with larger samples are necessary to draw conclusive interpretations. Those with early vs. late truncating variants were comparable across behavioral and cognitive measures. Cross-syndrome investigations that apply interdisciplinary methodological approaches are warranted to uncover the disease-causing processes in which epigenetic regulators of the histone machinery impact neurodevelopment (See Appendix Table 6).

## Data Availability

The data that support the findings of this study are available on request from the corresponding author. The data are not publicly available due to privacy or ethical restrictions.

## Appendix

**Table 6:** Variants identified in our clinical sample

Participant ID	Variant	Pathogenicity
Participant #1	c.3780delT and c.3782delC; p.P1261Lfs*33	Pathogenic
Participant #2	c.1285dupG; p.E429Gfs*7	Pathogenic
Participant #3	c.4228_4232delAAAGA; p.K1410Gfs*7	Pathogenic
Participant #4	c.1862 A > G; p.N621S; c.4952 C > T; p.P1651L	Variant of uncertain significance (VUS)
Participant #5	c.3385 C > T; p.R1129*	Pathogenic
Participant #6	c.4312G > T; p.E1438*	Pathogenic
Participant #7	c.5645_5646delTT; p.V1882Gfs*20	Pathogenic
Participant #8	c.3212 A > C; p.E1071A	Likely pathogenic
Participant #9	c.3661G > T; p.E1221*	Pathogenic
Participant #10	c.4228_4232delAAAGA; p.K1410Gfs*7	Pathogenic
Participant #11	c.4108G > T; p.E1370*	Pathogenic
Participant #12	c.1146_1147insG; p.Y383Ifs*14	Pathogenic
Participant #13	c.5212G > A; p.D1738N	Likely pathogenic
Participant #14	c.3070 C > T; p.R1024*	Pathogenic
Participant #15	c.3830_3831insTT; p.P1277Lfs*18	Pathogenic
